# Consumer perceptions and reported wild and domestic meat and fish consumption behavior during the Ebola epidemic in Guinea, West Africa

**DOI:** 10.7717/peerj.9229

**Published:** 2020-06-10

**Authors:** Lucie Duonamou, Alexandre Konate, Sylvie Djègo Djossou, Guy Apollinaire Mensah, Jiliang Xu, Tatyana Humle

**Affiliations:** 1 School of Ecology and Nature Conservation, Beijing Forestry University, Beijing, China; 2 Applied Ecology Laboratory, Faculty of Agronomic Sciences, University of Abomey-Calavi, Benin; 3 Department of Agroforestry, Institute Superior of Agronomy and Veterinary of Faranah (ISAV/F), Faranah, Guinea; 4 Department of Zoology/Primates Conservation Biology, Faculty of Science and Technology, University of Abomey-Calavi, Abomey-Calavi, Benin; 5 Agricultural Research Center of Agonkanmey, National Institute of Agronomic Research (INRAB), Abomey-Calavi, Benin; 6 Durrell Institute of Conservation and Ecology, School of Anthropology and Conservation, University of Kent, Canterbury, United Kingdom

**Keywords:** Bats, Pan troglodytes verus, Consumer behavior, Ebola virus disease, Guinea, Bushmeat, Public health, Food security, Zoonosis

## Abstract

The handling, capturing, butchering, and transportation of wildmeat can increase the risk of zoonoses, including the Ebola virus disease (EVD). Guinea, West Africa, experienced a catastrophic outbreak of EVD between 2013 and 2016. This study aimed to understand local people’s sources of information concerning EVD, their perceptions of potential wildlife carriers of EVD and their meat and fish consumption behavior during this period. A semi-structured questionnaire was administered to 332 participants in two urban centers (*N* = 209) and three villages (*N* = 123) between January 3 and March 30, 2015 in the prefecture of Lola in southeastern Guinea. Chi-square analyses revealed that, in rural areas, awareness missions represented the main source of information about EVD (94.3%), whereas in urban settings such missions (36.1%), as well as newspapers (31.6%) and radio (32.3%) were equally mentioned. Bats (30.1% and 79.4%), chimpanzees (16.3% and 48.8%) and monkeys (13.0% and 53.1%) were the most commonly cited potential agents of EVD in both rural and urban areas respectively, while the warthog (2.3% rural and 6.5% urban), crested porcupine (1.7% rural and 10.7% urban), duiker (1.19% rural and 2.6% urban) and the greater cane rat (1.1% rural and 9.5% urban) were also cited but to a lesser extent. However, 66.7% of rural respondents compared to only 17.2% in the urban area did not consider any of these species as potential carriers of the Ebola virus. Nonetheless, a fifth of our respondents reported not consuming any of these species altogether during the EVD outbreak. Among all seven faunal groups mentioned, a significant reduction in reported consumption during the Ebola outbreak was only noted for bats (before: 78.3% and during: 31.9%) and chimpanzees (before: 31.6% and during: 13.5%). Automatic Chi-Square Interaction Detection (CHAID) analysis revealed that the belief that bats or chimpanzees were associated with EVD or not had a significant effect respectively on their non-consumption or continued consumption. However, only 3.9% of respondents reported shifting to alternative protein sources such as domestic meat or fish specifically to avoid EVD. Only 10.8% reported consuming more domestic meat during the EVD outbreak compared with before; affordability and availability were the main reported reasons for why people did not consume more domestic meat and why two thirds reported consuming more fish. While increased domestic meat consumption was linked to the belief that duikers, the most commonly consumed wildmeat before the epidemic, were associated with EVD, increased fish consumption was not predicted by any EVD related factors. Our study revealed deep-rooted false beliefs among rural respondents and constraints when it comes to access to alternative protein sources such as domestic meat. Our findings emphasize the urgent need for greater consideration of the relationship between socio-economic context, food security, and public health.

## Introduction

Wildmeat is traditionally the main source of protein and an important revenue generator in sub-Saharan Africa (Fa, Ryan & Bell, 2005; Nasi et al., 2008; Van Velden, Wilson & Biggs, 2018). It is indeed an important nutritional, economic and cultural component of the livelihoods of rural communities in West and Central Africa, especially where alternative sources of protein are not customarily consumed on a regularly basis, and may be sparse and/or less affordable (Brown & Davies, 2007; Chausson et al., 2019). Hunting and bushmeat trade constitute a commercial activity valued at several billions of dollars annually in developing countries (Brashares et al., 2011; Robinson & Bennett, 2000) and the urban demand for wildmeat has increased in recent decades across many African countries (Barnes, 2002). In Central Africa, more than three million tons of wildmeat are harvested each year (Wilkie & Carpenter, 1999; Fa, Peres & Meeuwig, 2002). Previous studies have estimated that more than 900,000 kilograms of bushmeat are sold each year in Nigeria, and about150 million USD are obtained from the marketing of bushmeat in Côte d’Ivoire. (Fa, Peres & Meeuwig, 2002). It is also estimated that about five tonnes of bushmeat are transported from Africa to Europe each week (Chaber et al., 2010). In addition to urban demand, the increase in economic needs and the lack of alternative sources of revenue and protein are key factors driving the wildmeat trade (Cawthorn & Louwrens, 2015). The bushmeat trade is currently considered as one of the greatest threats to biodiversity because of its economic importance and its spatial scale (Ripple et al., 2016). In addition, the ease of access to previously remote areas, the availability of modes of transport such as motorcycles, and the use of guns are some of the factors that have transformed the traditional behavior of hunters and enhanced the commercialization of wildmeat, especially from rural to urban areas (Brashares et al., 2011; Fa et al., 2009). Uncontrolled hunting often leads to unsustainable harvesting of wildlife with disastrous consequences for biodiversity, ecosystems services and the livelihoods they support (Brashares et al., 2011).

The intense harvesting of wild fauna also increases the risk of exposure and transmission of zoonotic diseases and represents a serious risk to public health (LeBreton et al., 2012; Wolfe et al., 2005). People across West Africa are supplied with wildmeat through informal chains linking rural areas to urban centers (Brashares et al., 2011). That is why, today, hunting and the bushmeat trade constitute the main path for human exposure to zoonotic agents coming from wild animals, especially where people depend on wildmeat as their main source of protein and income (Kurpiers et al., 2016; Van Vliet et al., 2017). The simian immunodeficiency virus, human T-cell lymphotrophic virus, simian foamy virus, monkey pox virus, Ebola and Marburg filoviruses, anthrax, herpes viruses, hepatitis viruses, paramyxovirus and various parasites andbacteria (*Campylobacter* spp. and *Salmonella* spp) are some of the pathogens transmissible to humans through the handling, capturing, butchering, transportation and/or consumption of wildmeat (LeBreton et al., 2006; Van Vliet et al., 2017). Some of the most commonly cited potential reservoirs of zoonotic pathogens in Africa include bats and rodents, as well as non-human primates (Van Vliet et al., 2017). Among animal species carrying zoonotic viruses, bats have received particular attention during epidemics of zoonotic corona-, filo- and paramyxoviruses (Chomel, Belotto & Meslin, 2007; Plowright et al., 2015).

The health risks associated with the bushmeat trade are a growing concern worldwide. However, only a few studies have examined how risk perception of health risks influence people’s hunting, butchering and wildlife consumption habits. LeBreton et al. (2006) revealed that, in southern Cameroon, people’s perception of risk was not associated with hunting or consumption but rather the butchering process, which, in effect, is the riskiest activity when it comes to zoonoses. In addition, among a rural hunting community in Nigeria, although more than half of the hunters perceived zoonotic risks associated with hunting wildlife, only about a quarter adopted precautionary measures to minimize these risks (Friant, Paige & Goldberg, 2015). However, in a study carried out in Ghana, Lawson et al. (2017) revealed that disease risk perception associated with bats was relatively low. These studies and others generally argue the critical value of understanding risk perceptions and improving health-risk education to minimize zoonotic disease risk and future disease outbreaks.

The EVD outbreak that originated in Guinea recorded more than 28,000 EVD cases and 11,000 EVD-related deaths across 10 countries (WHO, 2016). During this EVD outbreak, the Guinean government and various health and conservation NGOs organized awareness campaigns to curtail the transmission of this virus (Faye et al., 2015). Despite the efforts of the government, the population, and national and international NGOs, the Ebola epidemic persisted until 2016, with significant consequences for many families in the region (Roemer-Mahler & Rushton, 2016). Political instability, poverty, poor health infrastructure, high population densities and a lack of information on Ebola, were some of the factors recognized to have specifically contributed to the spread and magnitude of the EVD epidemic in West African (Calnan et al., 2018). Several observers indeed recognized the lack of equipment in hospitals but also the low number of health professionals, especially in rural areas (Calnan et al., 2018). In addition, for many people, Ebola was not a natural disease but rather a laboratory-borne disease; unrelated to wildlife. This belief led to mistrust and several violent clashes between people and organisations in Guinea (Bedford, 2015). People’s beliefs and perceptions were hence critical obstacles to a rapid control of the epidemic in the country (Alexander et al., 2015).

Aside from focusing on the risks associated with human-human transmission, one of the messages conveyed was to avoid the consumption and trade of wild animals such as bats, monkeys, rodents, duikers, and chimpanzees (Loungou, 2017). These awareness campaigns led to a significant reduction in the presence of bushmeat on market stalls in urban areas in Guinea such as Guéckédou, the epicenter of the disease in southern Guinea, and Conakry, the capital city (Ly, 2017; WHO, 2015). However, in other localities of the country, especially rural areas, bushmeat was still consumed despite the potential risks involved (WHO, 2015). In the forest region of Guinea, due to the high price and limited availability of livestock meat, most ethnic groups continued to consume more wildmeat than livestock meat (Migliani et al., 2016a). As a result, medical teams, government and NGO officials were not always effective in their public health messaging and at discouraging the consumption of wildmeat in these areas (Suk et al., 2016). Our study focused on a district in southeastern Guinea, i.e., the forest region of Guinea, and aimed at better understanding and contextualizing differences between rural and urban areas and improving public health response and consideration of food security dimensions during disease epidemics such as EVD. The present study specifically aimed to better understand people’s sources of information concerning EVD, i.e., public health messaging platforms, between rural and urban areas during the outbreak. We also aimed to evaluate people’s perceptions of potential wildlife carriers of EVD and compare these between rural and urban areas. Finally, we sought to understand participants’ reported meat and fish consumption behavior during the EVD outbreak, and evaluate any reported changes with respect to respondents’ perceptions of potential wildlife carriers of EVD.

## Material and Methods

### Study area

**Figure 1:**
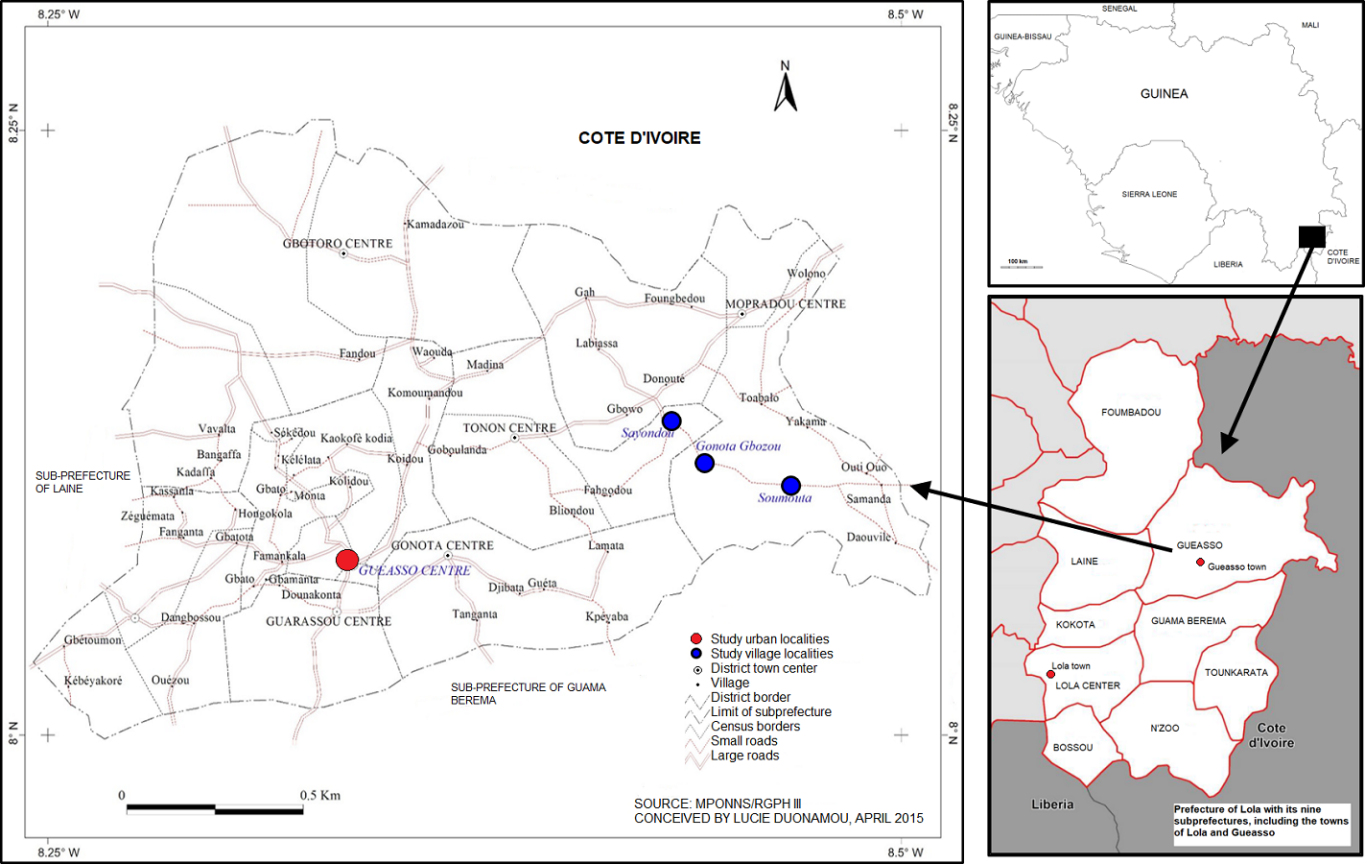
Maps indicating the location of Lola and of the Gueasso district highlighting the town of Gueasso and the three villages that were included in the study.

Lola Prefecture is located in the southeast of the Republic of Guinea between 7°32′ and 8°13′ north latitude, then 8°03′ and 8°35′ west longitude. With an area of 4,688 km^2^, it represents 1.9% of the national territory and 10.4% of the administrative region of Nzérékoré (PGRR-PZ, 1999). The population of the prefecture of Lola numbers 175,213 inhabitants including 83,286 men and 91,927 women with an average density of 37 inhabitants per km^2^ (DDPL, 2013). Gueasso, one of seven districts in the prefecture of Lola is bordered to the east by Côte d’Ivoire, to the west by Kokota district, to the north by the Foumbadou district, and to the south by Gama Bèrèma district (Fig. 1) (SPDL, 2012). It is located 60 km from Lola center and covers 975 km^2^ with 10,572 inhabitants, including 5,215 women according to the 2013 census (Migliani et al., 2016b).The villages of Gonota-Gbozou, Sayota, and Soumouta belong to the Gueasso district. They are located 25, 23 and 26 km^2^ from Gueasso connected by an untarmacked road network and have a total population of 304, 198 and 180 respectively (SRPD, 2010).

The area consists of numerous plains, hills, and plateaus ranging from 500-1,000 m in altitude. It has a tropical and sub-equatorial climate characterized by two seasons: a dry season lasting 3 to 4 months (December to March) and a rainy season spanning 8 to 9 months (April to November) of the year. The average annual rainfall is 3,000 mm, and an average temperature of 24 °C (DDPL, 2013). The main socio-economic activities include agriculture, hunting, fishing, crafts and local trade (DDPL, 2013). The local languages in the Lola urban center and Gueasso district are Zogwota, Maouka, Koniaké, Kônon, Manon, Koniaké and French, which is the official national language of Guinea. The religions practiced in the area are Christianity, Islam, and Animism (SPDL, 2012).

The vegetation in the district is tropical and sub-equatorial, but degraded by anthropogenic activities, including agriculture and illegal timber exploitation that have dramatically reduced the natural vegetation cover formed by dense forest patches (Suk et al., 2016). The faunal species in the region are dominated by the greater cane rat (*Thryonomys swinderianus*), the buffalo *(Kobus ellips iprymnus)*, the green monkey *(Cercopithecus aethiops*), the yellow-backed duiker *(Cephalophus sulvicultor),* the warthog *(Phacochoerus africanus)*, the bushbuck *(Tragelaphus scriptus)*, the crested porcupine *(Histrix cristata)*, the African brush-tailed porcupine *(Atherurus africanus)*, the giant pangolin *(Smutsia gigantea)*, the West African crocodile *(Crocodylus suchus)*, the leopard (*Panthera padus*) and numerous bat species (PGRR-PZ, 1999). Some species, such as the critically endangered chimpanzee *(Pan troglodytes verus*), have declined in recent years, and deforestation and hunting are the main drivers of wild species decline in the locality (Suk et al., 2016).

The choice of Gueasso district was based mainly on the presence of the Ebola epidemic and the high levels of hunting activity in the prefecture (SRPD, 2010). The selection of the villages of Gonota-Gbozou, Sayota, and Soumouta was based on the known presence of hunting in these localities for both subsistence and commercial purposes (L. Duonamou, 2013-2016, pers. obs.). The urban centers of Lola and Gueasso were selected because of the presence of a bushmeat market associated with a high demand for wildmeat (Migliani et al., 2016a).

### Research design and data collection

This study was reviewed and approved by the ethics’ committee of the University of Abomey Calavi, Republic of Benin. The Ministry of the Environment, and Water and Forest authorities in Guinea granted permission to pursue this research and this study in Gueasso, Prefecture of Lola, Guinea. This study complied with all legal requirements for research within the country. The objectives and process of the study were explained to the administrative and local authorities, as well as to the participants before starting in each locality. We delivered the questionnaire individually face to face. Individual participation was secured through verbal consent and was voluntary and anonymous.

For the purpose of this study, we randomly selected participants, ensuring a single respondent per household. While we approached every single household in the villages, we randomly sampled households around the center of the main towns of Lola and Gueasso. Among the 351 households approached, 332 (mean age ± 1 SD = 44 ± 12.45) accepted to partake (94.6% overall response rate), i.e., 209 (131 men and 78 women) in urban and 123 (84 men and 39 women) in rural areas (Table 1). Across all areas, both rural and urban, we interviewed 215 men (mean age ± 1 SD = 47 ± 12.30) and 117 women (mean age ± 1 SD = 38 ± 10.87) (Table 1). The urban response rate was slightly higher (97.2%) than for the rural sample (90.5%). We finally secured the participation of 42 people in Gonota-Gbozou, 41 in Soumouta and 40 in Sayota and 110 and 99 individuals from different households in the town sof Lola and Gueasso respectively (Table 1).

**Table 1:** Number of respondents across localities by gender and primary occupation.

Primary occupation	Urban areas						Rural areas								
	Lola			Gueasso			Gonota-Gbozou			Soumouta			Sayota		
	Men	Women	Total	Men	Women	Total	Men	Women	Total	Men	Women	Total	Men	Women	Total
Farmers	40	0	40	40	0	40	20	0	20	20	0	20	20	0	20
Hunters	15	0	15	20	0	20	10	0	10	5	0	5	5	0	5
Forest service agents	2	0	2	3	0	3	0	0	0	0	0	0	0	0	0
Community leaders	2	0	2	2	0	2	0	0	0	0	0	0	0	0	0
Local restaurant owners	0	20	20	0	5	5	0	0	0	0	0	0	0	0	0
Bushmeat sellers	0	0	0	0	3	3	0	0	0	0	0	0	0	0	0
Community elders	4	0	4	3	0	3	2	0	2	1	0	1	1	0	1
Housewives	0	27	27	0	23	23	0	10	10	0	15	15	0	14	14
TOTAL	63	47	110	68	31	99	32	10	42	26	15	41	26	14	40

One of the purposes of the questionnaire was to investigate respondents’ opinions of wildlife potentially posing a risk for EVD transmission to humans. Albeit the diversity of fauna in the Lola Prefecture, we focused our study on seven wildlife taxa (bat, monkey, crested porcupine, greater cane rat, duiker, chimpanzee and warthog) cited by researchers (e.g., Leendertz et al., 2017) and awareness-raising NGOs during the epidemic as potential carriers of the Ebola virus. The questionnaire also aimed to capture respondent-level basic demographic information (age, gender, primary occupation, ethnicity, religion), their main sources of information concerning EVD, and their meat, i.e., wild and domestic, and fish consumption behavior before and during the EVD outbreak (File S1). We did not set a timeframe for consumption of the focal wildlife taxa before the EVD outbreak since we wanted to establish if respondents previously consumed them at all, i.e., they were not averse to their consumption, and did not hold any taboos against their consumption. The study also explored the reasons for why respondents reported consuming more or less domestic meat or fish during the EVD epidemic. Depending on the respondent’s primary occupation, the set of questions were slightly different although the core set of questions were the same across all participants. All questions were open-ended (File S1).

Data collection was conducted between January 3rd and March 30th, 2015 in the Lola prefecture. The questionnaire was translated into all local language and where possible was delivered in French which is the official national language of Guinea. The first author is fluent in French, Guerzé, and Konon and delivered the questionnaire whenever possible, else she was assisted by local assistants.

### Data analysis

All data were analyzed using IBM SPSS Statistics v.25. Firstly, we used chi-square tests to examine the association between formal sources of information about the Ebola virus and the interviewees’ perceptions of what wildlife were associated with EVD between rural and urban areas, as well as reported consumption or non-consumption of wildmeat before and during the EVD outbreak. Z scores were used for post hoc analysis of the results to identify which cells within the contingency tables were significant, with adjusted standardized residuals of >—1.96— being significant at the 5% level. Secondly, we analyzed predictors of continued or non-consumption of individual faunal groups; independent variables included age-class (20–39 years old and >40 years old), gender, religion, rural versus urban, occupation, perceptions of faunal groups acting as potential carriers of EVD, reported increase in livestock meat or fish and sources of information concerning EVD. Bats and chimpanzees were the focus of this analysis since they were the most cited species in the media and by our respondents with respect to the risk of zoonotic transmission in the context of EVD. Although monkeys were also frequently cited, we could not unfortunately carry out this analysis for monkeys because, although all faunal groupings had distinct vernacular names, the term ‘monkey’ could in some instances also include ‘chimpanzees’. It is also for this reason that the variable ‘belief that monkeys were potential vectors or carriers of EVD’ was also excluded from these analyses. For the purpose of this analysis, we used a Chi-squared Automatic Interaction Detection (CHAID), a decision-tree data-mining method useful in examining the importance of the above categorical independent variables and the combination that best predicted individual reported non-consumption or continuation of consumption of bats or chimpanzees during the EVD outbreak (Gorunescu, 2011). We also employed a CHAID analysis to examine the influence of the same categorical variables on the reported increase in the consumption of livestock meat or fish during the EVD outbreak. All statistical significance was set at *p* < 0.05.

## Results

### Socio-demography information

Of the 332 urban and rural respondents, 61.7% were >40 years old (54.5% in rural areas and 66.0% in urban centers), and 93.97% were married, 4.51% single and 1.50% were divorced. More than half of the respondents (55.7%) declared themselves as Animists, while 28.6% and 15.7% Christian and Muslim respectively. The ethnic mix of our sample was comprised of Konon (33.1%), Zogwota (20.5%), and Guerzé (18.4%) Koniaké (9.0%), Manon (8.4%), Maouka (6.9%), Malinké (2.1%), Toma (1.2%) and Kissi (0.3%). Respondents included farmers (42.2% (*N* = 140)), housewives (26.8% (*N* = 89)), hunters (16.6% (*N* = 55)), and community elders (3.3% (*N* = 11)) in both rural and urban localities, in addition to forest service agents (1.5% (*N* = 5)), community authorities, i.e., government officials (1.2% (*N* = 4)), local restaurant managers (7.5% (*N* = 25)), and bushmeat sellers (0.9% (*N* = 3)) who were only located in urban areas (Table 1).

### Formal sources of information concerning EVD

**Figure 2:**
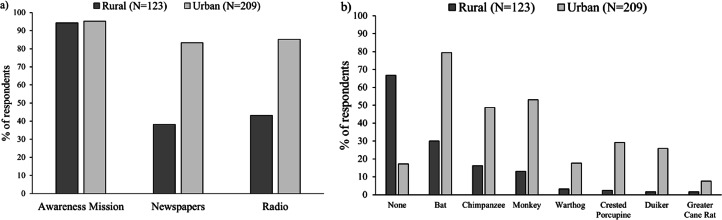
Percentage of respondents in both urban and rural areas who reported (A) use of different media channels of information concerning the Ebola virus disease; (B) perceiving specific species as a potential agent of the Ebola Virus Disease.

Respondents in both rural and urban areas only ever reported three main sources of information concerning EVD, i.e., radio, newspapers and awareness-raising missions by government and NGOs (Fig. 2). There was no significant association between main sources of information concerning EVD and the two towns, i.e., Lola and Gueasso (Chi-square test: *X*^2^(2) = 2.537, *p* = 0.281) and between main sources of information concerning EVD and the three villages (Chi-square test: *X*^2^(4) = 0.686, *p* = 0.953), justifying us to pool the data for the two urban centers and the villages together in order to compare urban versus rural areas. We found a highly significant association between sources of information concerning EVD and urban and rural areas (Chi-square test: *X*^2^(2) = 19.989, *p* < 0.001). Post hoc analysis revealed that in rural areas, people relied significantly less on newspapers and radio and significantly more on awareness missions than expected, whereas in urban settings all three channels were reported equally as main sources of public health messaging concerning EVD (Fig. 2A).

### Perceptions of potential wildlife vectors of the Ebola virus

Bats (30.1% and 79.4%), chimpanzees (16.3% and 48.8%) and monkeys (13.0% and 53.1%) were the most commonly cited potential agents of EVD in both rural and urban areas respectively. Other taxa, including the warthog (3.3% and 17.7%), the duiker (1.6% and 25.8%), the crested porcupine (2.4% and 29.2%) and the greater cane rat (1.2% and 9.5%) were also mentioned but to a lesser extent in both rural and urban areas respectively (Fig. 2B). Nevertheless, 66.7% (82/123) of respondents in rural areas compared to only 17.2% (36/209) in the urban area reported that they did not consider any of these species as potential carriers of EVD (Fig. 2B). There was, however, a significant association between reported EVD carrier taxa and rural and urban settings (Chi-square test: *X*^2^ (6) = 14.563, *p* = 0.024). Bats were reported significantly more than expected in rural areas than urban areas, while the reverse was true of the duiker and the crested porcupine that were reported significantly more than expected in urban areas than rural areas (Fig. 2B).

### Wildmeat consumption before and during the Ebola outbreak

**Figure 3:**
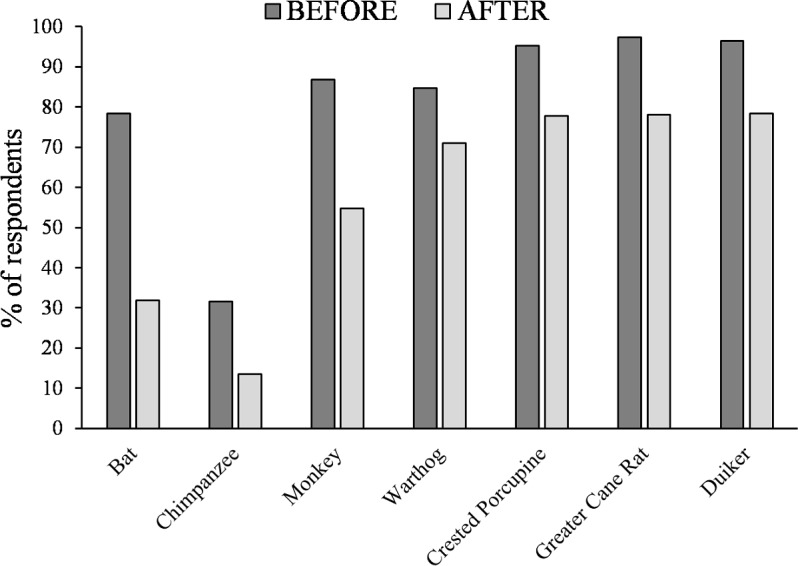
Percentage of respondents who reported consuming the seven mentioned potential faunal vectors of the Ebola Virus Disease before and during the Ebola outbreak.

The reported dominant wildmeat consumed before and during the EVD outbreak were duiker (96.4% and 78.3%), greater cane rat (97.3% and 78.0%) and crested porcupine (95.2% and 77.7%) in rural and urban areas respectively (Fig. 3). Nearly a fifth of all respondents (19.6%; 65/332) reported not having consumed any of the seven faunal groups mentioned as associated with EVD during the outbreak with only four respondents (3.2%, 4/123) reported doing so in rural areas, and 65 (31.1%, 65/209) in the urban centers of Lola and Gueasso. Generally, there was a decline in reported consumption of these seven faunal groups that respondents had reported as potential vectors or carriers of EVD before and during the Ebola epidemic (Fig. 3). There was indeed an overall significant shift in the reported consumption of faunal groups (Chi-square: *X*^2^ (6) = 44.622; *p* < 0.001) (Fig. 3). However, post hoc analysis revealed that differences in reported consumption before and during the EVD outbreak was significant only for bats (before: 78.3% and during the EVD outbreak: 31.9%) and chimpanzees (before: 31.6% and during the EVD outbreak: 13.5%), while the warthog was consumed significantly more than expected (before: 84.6% and during the EVD outbreak:71.1%). There was no significant association between these two periods in the reported consumption of the crested porcupine, the duiker, the greater cane rat, and monkeys.

### Predictors of reported non-consumption of bats during the EVD outbreak

**Figure 4:**
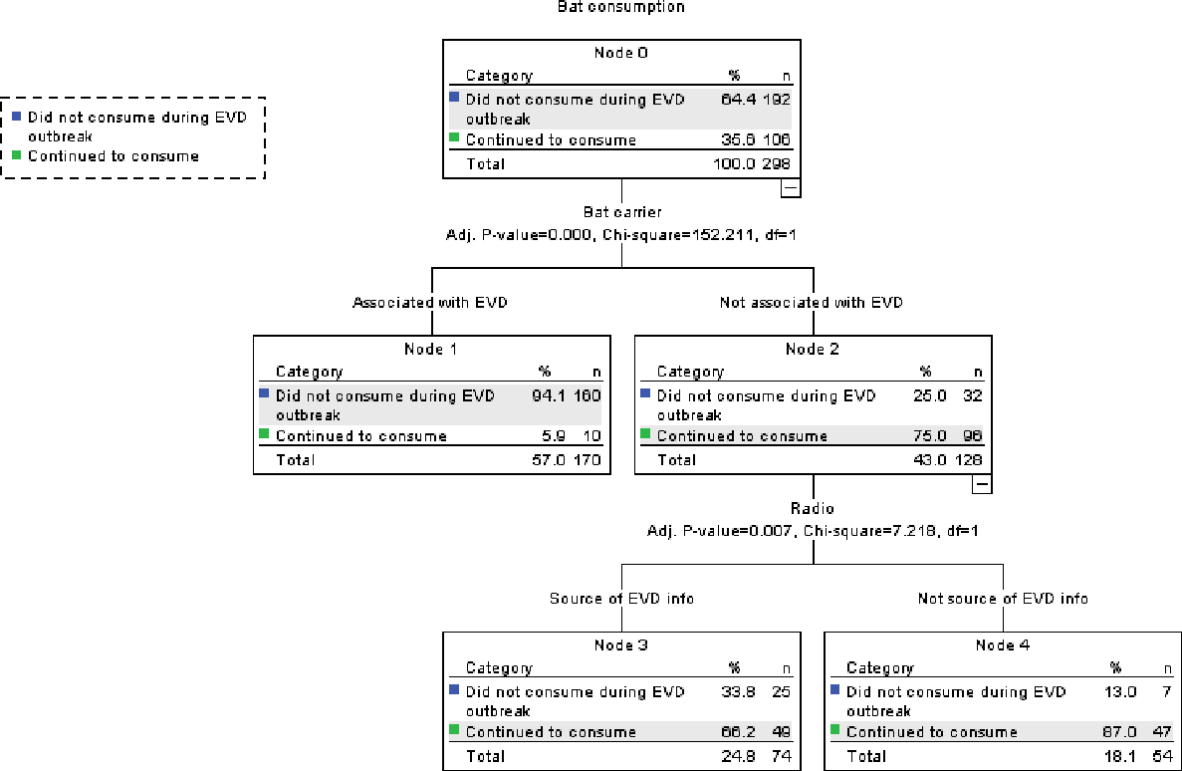
CHAID classification tree of predictors of bat consumption patterns during the EVD outbreak.

Two hundred and sixty respondents (78.3%, with 100% in rural areas and 83.7% in urban areas) reported consuming bats before the EVD outbreak. For the purposes of the CHAID analysis, those respondents who claimed to have never consumed bats before and during the EVD outbreak (*N* = 34) were excluded from the analysis. Whether respondents believed that bats were EVD carriers or not was a significant predictor of the reported non-consumption of bats during the EVD outbreak (Chi-square: *X*^2^ (1) = 129.43; *p* < 0.001) (Fig. 4). Among those who did not identify bats as posing a potential risk of EVD transmission, the CHAID analysis revealed nevertheless a significant effect of radio as a source of information concerning EVD, i.e., those who reported getting their EVD information from the radio were significantly less likely to consume bats than others (Chi-square: *X*^2^ (1) = 7.288; *p* < 0.001) (Fig. 4).

### Predictors of reported non-consumption of chimpanzee during the EVD outbreak

**Figure 5:**
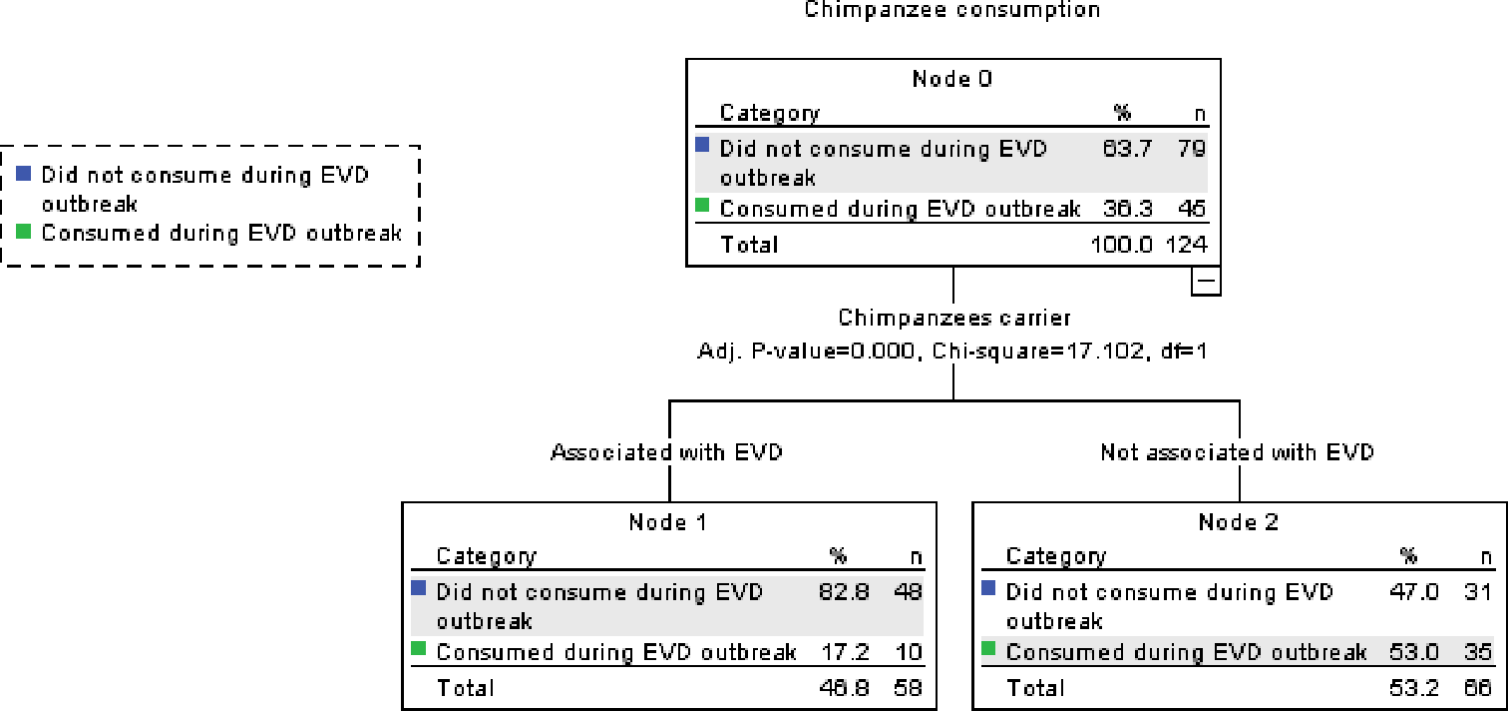
CHAID classification tree of predictors of chimpanzee consumption patterns during the EVD outbreak.

Hundred twenty-four respondents (31.6%%, with 12.2% in rural areas and 49.7% in urban areas) reported consuming chimpanzees before the EVD outbreak. For the purposes of the CHAID analysis, those respondents who claimed to never have consumed chimpanzees before and during the EVD outbreak (*N* = 208) were excluded from the analysis. Our analysis revealed that whether respondents believed that chimpanzees were EVD carriers or not was a significant and the main predictor in the reported non-consumption of chimpanzees during the EVD outbreak (Chi-square: *X*^2^ (1) = 17.102; *p* < 0.001) (Fig. 5).

### Predictors of increase in fish and livestock meat consumption during the EVD outbreak

All forest service agents and community leaders, i.e., government officials, (100%) reportedly consumed more livestock meat during the Ebola epidemic compared with before, while only 44% of restaurant owners, 27.3% of elders, 11.2% of housewives, 1.8% of hunters, 1.4% of farmers and 0% bushmeat traders reported doing so. In contrast, three quarters of the respondents reported consuming more fish during the EVD outbreak.

**Figure 6:**
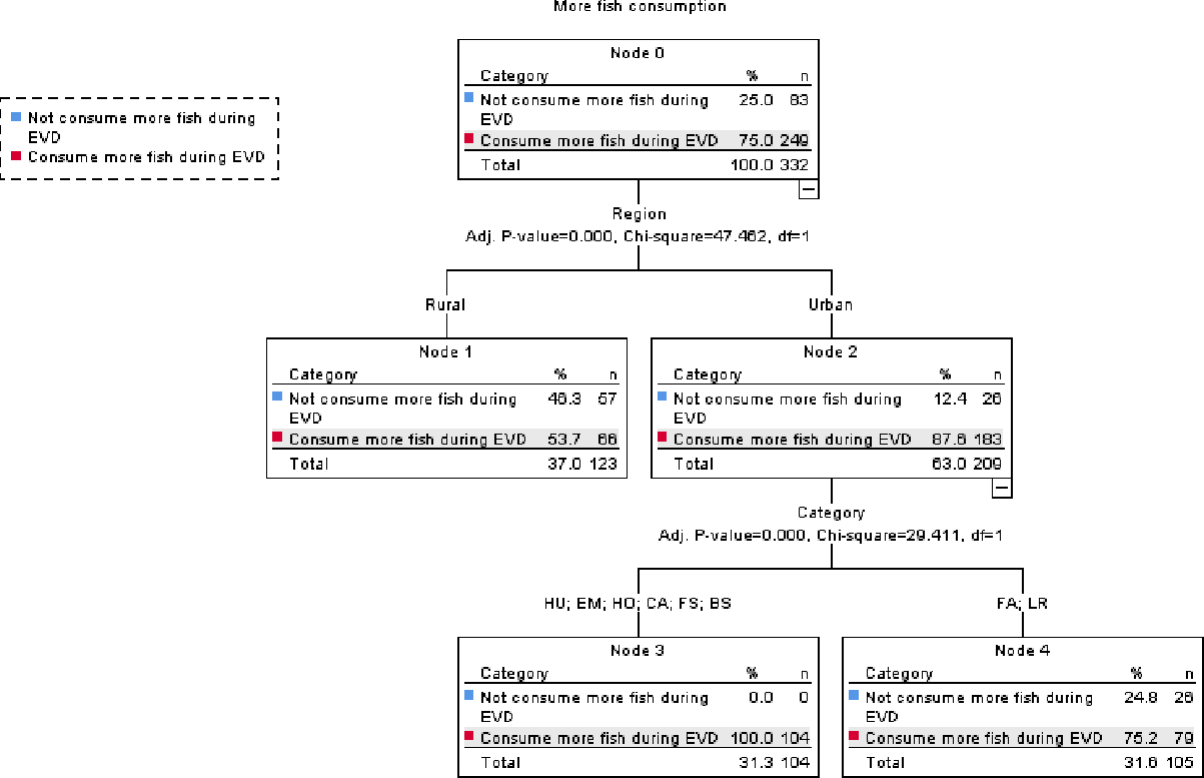
CHAID classification tree of predictors of increase in fish consumption during the EVD outbreak with ‘Category’ representing occupation and HU, hunter; EM, Village elder; HO, Housewife; CA, Community Authority; FS, Forest Service Agent; BS, Bushmeat Selle.

The CHAID analysis revealed a significant difference in fish consumption between rural and urban areas, whereby urban respondents reportedly consumed significantly more fish than rural respondents (*X*^2^ (1) = 47.462, *p* < 0.0001) (Fig. 6). In addition, in urban centers, occupation was a significant predictor of fish consumption with farmers and local restaurant managers consuming significantly less than respondents with other occupations (*X*^2^ (1) = 29.411, *p* < 0.0001) (Fig. 6). Among those respondents who reported consuming more fish, only 3.2% confirmed doing so to avoid EVD, 2.0% because wildmeat consumption was prohibited, 2.0% because they preferred to consume fish, 2.8% because there was a lack of bushmeat and the majority, i.e., 90.0%, because it was cheaper than alternatives such as domestic or wild meat.

**Figure 7:**
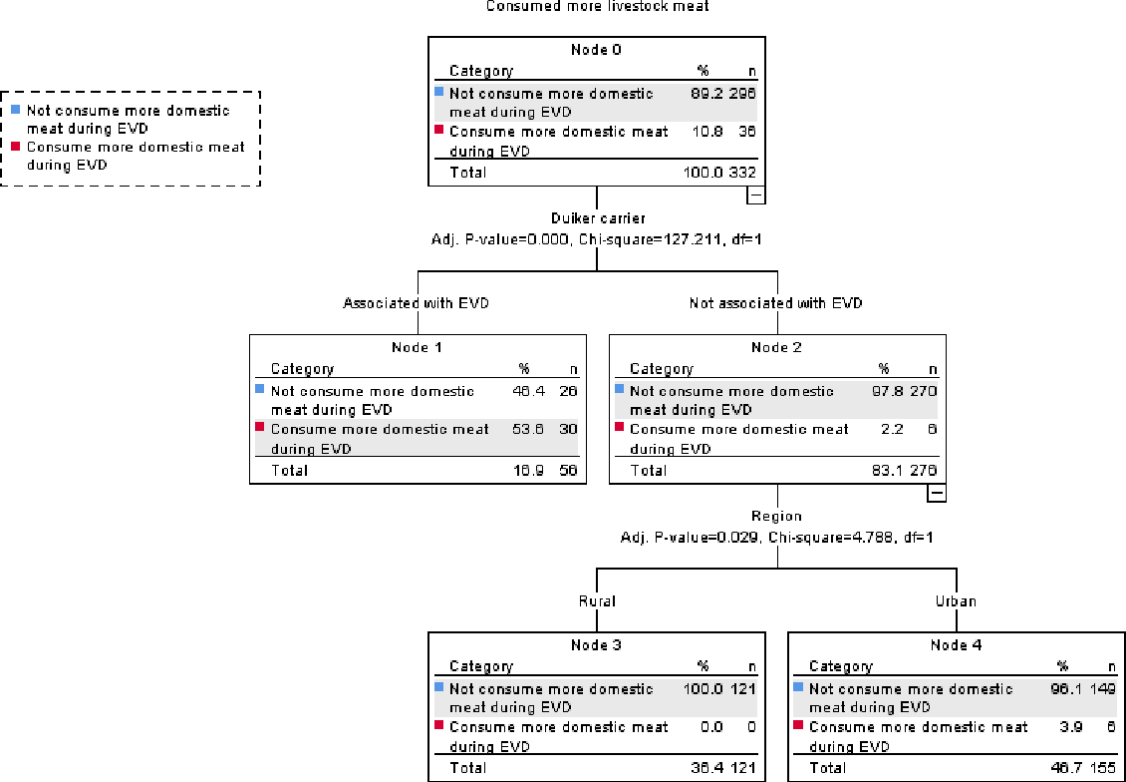
CHAID classification tree of predictors of increase in domestic meat consumption during the EVD outbreak.

Only 10.8% (36/332) of respondents reported consuming more domestic meat during the EVD outbreak; all of these respondents were from urban areas (Fig. 7). Belief that duikers are carriers of EVD was a major factor in predicting a reported increase in livestock meat consumption (*X*^2^ (1) = 127.211, *P* < 0.0001) (Fig. 7). However, among those respondents who did not associate duiker meat with EVD, significantly more reported consuming more domestic meat in urban areas than rural areas (*X*^2^ (1) = 4.788, *P* = 0.029) (Fig. 7). Among those who reported consuming more domestic meat, 47.2% reported doing so because wildmeat consumption was prohibited during the Ebola epidemic, 19.4% to avoid EVD, 27.8% because it was preferred by their customers, while 5.6% of the respondents consumed livestock meat more than before the epidemic because there was lack of bushmeat. The remainder of the respondents reported consuming less livestock meat since the EVD outbreak mainly because livestock meat was too expensive (68.3% in rural areas and 59.3% in urban areas), because it was too difficult to breed livestock (31.7% in rural areas and 18.7% in urban areas), or because they preferred bushmeat (0% rural areas and 4.8% in urban areas).

## Discussion

Our study highlighted significant differences in disease risk perception and channels of information about EVD between rural areas and urban centers. Rural people in our sample mainly received their information about EVD through awareness-raising missions in their village, while urban respondents reported gaining their information about EVD equally through newspapers, awareness-raising missions, and radio, the only reported formal channels from which information about EVD was reportedly acquired. None of our respondents mentioned television (TV) or social media as sources of information. Kuukyi, Amfo-Otu & Wiafe (2014) found that almost half of the respondents in urban areas in Ghana confirmed receiving their information about how the Ebola disease is transmitted from the radio. Similarly Keita (2015) revealed that school children in Bamako, Mali, reported hearing information about how to avoid Ebola mainly via the radio. These findings highlight the high value of radio as a medium for disseminating critical information to the general public in West African countries, especially in urban areas where people had greater access to radio and electricity and where levels of illiteracy may be high (Keita, 2015). Nevertheless, in our study area, only a few people in rural areas actually owned a TV or a radio, and even if they had one, only a few people listened to the radio or watched TV, especially during periods of agricultural activities, which corresponded to our study period. Some rural areas do not even have access to electricity to power televisions or a radio, and newspapers also rarely ever reach such locations (Duonamou pers. obs.). This explains why awareness-raising mission campaigns were the dominant formal mode of transmission of information about EVD in rural areas. In urban areas, all these various channels exist and different respondents therefore engaged with the multiple channels available.

Although all seven faunal groups were perceived to act as potential vectors of EVD, bats, chimpanzees and monkeys were the three most commonly mentioned. Our study revealed that bats and chimpanzees were reportedly consumed significantly less often during the Ebola epidemic period than before, while this was not the case for any of the other faunal groups mentioned. However, we must note that our pre- and post EVD design concerning wildmeat consumption was not balanced in its timeframe, since pre-EVD, respondents were simply asked whether the species was part of their dietary repertoire or not, and not whether they had recently consumed these species or not within the recent past. Some species such as the chimpanzee in particular which is now much rarer in the sub prefecture of Gueasso may not have been consumed recently due to its low availability, but given the opportunity, we assumed that people who consumed it in the past were more likely to consume it again. Nevertheless, our findings suggest that people’s belief that these species were potential carriers of EVD was a determining factor in predicting their non-consumption during the EVD outbreak; while neither religion, gender, occupation, location (rural versus urban dwellers), age-class, the perception of other faunal groups as potential EVD carriers or reported increase in domestic meat or fish consumption were significant predictors. There was also no influence of public messaging platforms on the non-consumption of chimpanzees. However, among those who did not perceive bats as carriers, those who listened to the radio tended to consume significantly less bats than others who did not, indicating that albeit their disbelief, a few respondents nevertheless adopted a cautionary approach to their wildmeat consumption habits based on media information secured via the radio in spite of whether they were urban or rural dwellers. The warthog was consumed significantly more than expected during the EVD epidemic, while other species, i.e., duiker, crested porcupine, greater cane rat and monkey, were consumed in relative similar proportions compared with before. These five faunal groups happened to be the ones most consumed before the EVD epidemic and are hence potentially preferred. It is also worthy to note that, among these faunal groups, monkeys, rodents and hogs are also well known for their crop-foraging habits (Garriga et al., 2018), which may have additionally influenced their continued hunting, consumption and commercialization in the region (Duonamou et al., in press). Although LeBreton et al. (2006) found that disease risk perception in Cameroon was only significant for wildmeat butchering rather than its consumption, our findings suggest that when it comes to EVD, the belief that a species could act as a carrier or a vector of EVD could also significantly influence people’s consumption behavior. This finding concurs with that of Ordaz-Németh et al. (2017) who noted a significant influence of the perceived risk associated with the consumption of bushmeat during the EVD outbreak on people’s eating habits in Liberia.

Nevertheless, two thirds of our respondents in rural areas versus about a quarter in urban areas perceived that none of the seven faunal groups mentioned as potential carriers or vectors of EVD posed any risk of zoonoses when it comes to EVD. These respondents tended to believe that EVD is not natural and blamed developed countries for its spread in Africa. Moulin (2015) also recorded such beliefs in the hardest-hit regions of Guinea and elsewhere in West Africa. Unfortunately, this confusion and misunderstanding caused doubts among rural people who hence mistrusted political decision-makers and healthcare workers (Dudas & Rambaut, 2014). Respondents with such beliefs therefore tended to maintain their bushmeat consumption habits and potentially mistrust information conveyed during the course of awareness raising missions or via other more formal sources of information such as the radio. During the 1995 Ebola epidemic in Côte d’Ivoire, people also continued to consume bushmeat during the epidemic (Caspary, 1999). In their study in Liberia, Ordaz-Németh et al. (2017) similarly found that duiker, pangolin or monkey meat consumption did not change during the EVD outbreak. Bonwitt et al. (2018) in Sierra Leone similarly revealed that the proffered ban on wildmeat consumption and the strong messaging around the health risks associated with wildmeat had little effect as it contradicted people’s own safe experiences with wildmeat consumption. Nonetheless, in our study, although most respondents continued to consume wildmeat during the EVD outbreak, the presence of EVD did significantly influence people’s wildmeat consumption habits of bats and chimpanzees during the EVD outbreak. In addition, a fifth of our respondents (*N* = 65), mostly from urban areas, reported not having consumed any of the seven faunal groups mentioned as potential vectors or carriers of EVD during this period.

However, only a minority of respondents claimed outright to have increased their fish or domestic meat consumption in order to avoid EVD. Among those few respondents who reported consuming more domestic meat during the EVD outbreak, nearly half did so because wildmeat consumption was prohibited by law. While more than half of the respondents who believed that duikers were potential carriers of EVD reported consuming more livestock meat, nearly all who did not hold such a belief did not report consuming more domestic meat during the EVD outbreak. People with regular monthly salaries and employed by the government, i.e., forest service agents and community leaders, reportedly consumed more livestock meat during the EVD outbreak than people with other primary occupations that potentially generate less and more irregular incomes. This result contrasts with Ordaz-Németh et al. (2017)’s study in Liberia which found that wealthier people showed a less pronounced reduction in bushmeat consumption during the EVD outbreak. Among the 89.2% of the respondents who reported consuming less livestock meat during the EVD epidemic, 70.3% did so because they claimed it was too expensive. Price was therefore in our study sample an evident deterrent to consuming domestic meat as a fallback source of protein to avoid potentially consuming wildmeat. This result concurs with findings in other rural and urban centers in Guinea, such as Faranah and Kouroussa and surrounding villages, where the price of domestic meat is prohibitively high compared with bushmeat or fish (Duonamou et al. unpub. data). These findings contrast significantly with that from other urban centers, particularly in Central Africa, where bushmeat is a luxury item and therefore less accessible to poorer households (Chausson et al., 2019; Cowlishaw, Mendelson & Rowcliffe, 2005). In addition, many respondents raised the issue of the difficulties associated with raising livestock, which is a factor that also possibly constrained availability and affected supply and affordability, especially in rural areas. In the Republic of Congo, domestic species breeding is very limited in certain areas, since many species are unable to survive environmental conditions and are often prone to disease (Ofouémé-Berton, 1993). To meet their needs, rural populations in sub-Saharan African, including in our study area, hence relied on wildmeat, as well as fish, and potentially other sources of protein such as insects, caterpillars, larvae, and snails (Lindsey et al., 2012). Our study revealed that bushmeat consumption is not just a matter of habit or preference as only a few respondents reported during our study, but also of convenience as wildmeat is more readily accessible, free or cheaper than livestock meat, especially in rural areas. Our findings enhance findings from elsewhere in Africa that show that food security, wildlife conservation, and public health are highly interconnected (Friant et al., 2020).

In light of the ban on bushmeat consumption, fish and edible mushrooms were the most important and invaluable sources of protein in Côte d’Ivoire during the Ebola crisis (Dindé et al., 2017). In our study, three quarters of the respondents reported consuming more fish during the EVD outbreak compared with before, although this pattern was more significant in urban areas than rural areas. However, in stark contrast with Dindé et al. (2017)’s findings, only 5.2% of our respondents reported prohibition of wildmeat consumption or avoidance of EVD as their main reasons for consuming more fish. During our study, fish was abundantly available and cheap as the period coincided with the peak-fishing season and urban areas additionally benefitted from coastal supplies of dried or frozen marine fish into city markets (Duonamou pers. obs.). Affordability and availability were hence the main reasons for the reported increase in fish consumption, rather than fish acting as a protein substitute for wildmeat due to perceived health risks or the government ban on wildmeat consumption. Nevertheless, our study indicates that fish, which can be sold in many forms and at various prices, could be a good alternative to wildmeat in many areas and could help address the nutritional challenges faced by many households during major disease epidemics while minimizing the potential risks of exposure to zoonoses. In their study in Ghana, Brashares et al. (2004) revealed a direct relationship between fish supply and wildmeat demand, arguing consequently that improving fisheries management could help offset demand for bushmeat. However, this would also require systematic monitoring of fishing permits and an enforcement of fishing regulations to ensure sustainability and an increase in the availability of fish on both rural and urban markets at affordable prices (Brashares et al., 2004; Jacobs et al., 2016; Junker et al., 2015; Rentsch & Damon, 2013).

## Conclusions

Diseases such as the Ebola Virus Disease cannot only affect wildlife species, but also critically threaten the life, as well as the nutritional and health security of people in affected countries. Despite the EVD outbreak and a government ban on the consumption of wildmeat, our study revealed that wildmeat consumption still persisted, albeit a marked reduction in the consumption of bats and chimpanzees during the EVD outbreak, which was significantly predicted by respondents’ disease risk perception. However, albeit wide exposure to EVD awareness-raising missions, the majority of respondents in rural areas did not believe that wildlife could act as vectors of EVD. Future awareness-raising campaigns need to tread carefully when developing their messaging especially in rural areas where other beliefs may prevail as to the origin of the Ebola virus epidemic and people may under-appreciate the risk associated with the handling, capturing, butchering, and transportation of infected carcasses and where many households are highly dependent on wildmeat for protein and income, and hunting to protect their crops. Our findings also highlighted the urgent need to address the hunting of critically endangered species such as the western chimpanzee, whose capture and hunting are prohibited by law (Humle et al., 2016), regardless of the EVD epidemic and whose consumption was reported among 13.6% our respondents during the EVD epidemic in this region of Guinea, primarily in urban centers. Although the belief that duikers could act as potential carriers or vectors of EVD significantly influenced people decision to increase their livestock consumption, very few of our respondents shifted their protein consumption habits from wildmeat to other sources such as livestock and/or fish to minimize the risks of EVD transmission or in response to prohibitions on wildmeat consumption. To minimize disease-risk exposure from wildmeat, there is an urgent need, especially for rural communities, to gain access to alternative favored, affordable and sustainable sources of protein and technical and veterinarian assistance for raising livestock. In addition, it is also urgent to address co-existence issues with species that incorporate crops into their diet to minimize this major driver of wildmeat hunting and trade (Duonamou et al., in press). Such factors, other than addressing rumors and disbeliefs concerning EVD via effective and culturally-sensitive awareness raising campaigns, especially via radio and awareness raising missions, are essential to reduce the vulnerability of people in the region to future risks of zoonosis of EVD and potentially other emerging pathogens, associated with the handling, capturing, butchering, and transportation of infected wildlife carcasses. Finally, our findings highlight the potential greater value of bottom-up interventions in developing countries, such as public health messaging, behavior change campaigns and local promotion of favored and affordable alternative protein resources, in contrast to top-down interventions such as regulations imposing bans and/or restrictions on people’s behavior.

##  Supplemental Information

10.7717/peerj.9229/supp-1 File S1 Data collection questionnaire

10.7717/peerj.9229/supp-2 File S2 Dataset

## Additional Information and Declarations

### Field Study Permissions

The following information was supplied relating to field study approvals (i.e., approving body and any reference numbers): The Ministry of the Environment, and the animal conservation authorities in Guinea granted permission to pursue this research and this study complied with all legal requirements for research within the country.

### Data Availability

The following information was supplied regarding data availability: The raw data are available in the Supplemental Files.
