# Supplementary material for: Consumer perceptions and reported wild and domestic meat and fish consumption behavior during the Ebola epidemic in Guinea, West Africa
Source: PeerJ. 2020 Jun 10;8:e9229. doi: 10.7717/peerj.9229 (PMC7293194; doi:10.7717/peerj.9229)
Supplement: File S1 [file peerj-08-9229-s001.docx]

**Questionnaire**

Sheet N ° / ____ / Date of investigation / __ / __ / __ / Investigator: / _____ / Interpreter /___/

|  | Responses |
| --- | --- |
| Prefecture |  |
| Sub-prefecture |  |
| District |  |
| Village |  |

**Profile of the respondents**

|  | **Choice of responses** | **Responses** |
| --- | --- | --- |
| Age |  |  |
| Gender | 1 =Male; 2= Female |  |
| Ethnicity |  |  |
| Religion | 1 =Traditional; 2 =Christian ; 3= Muslim 4= Other |  |
| Marital status | 1= Single, 2 =Married, 3 =Widowed / Widowed;  4= Separated / Divorced |  |
| Occupation |  |  |

1. **Hunters**

| **Questions** |
| --- |
| What are the main species of fauna do you encounter here? |
| Which animal species are hunted locally? |
| Are there some species that people avoid hunting? Which ones? |
| Which species do people tend to prefer to consume locally? |
| In your opinion, what animal species in this area have become rare or extinct? |
| In which vegetation type do people typically hunt locally? |
| Do you own livestock? Which one (s)? |
| What difference do you make between the price of bushmeat and that of livestock meat? Why this difference? |
| How do you appreciate the current wildlife population compared with 5 years ago? |
| What is the most abundant wildlife species in this community nowadays? |
| Do you know of any species that are now rare or have disappeared from this area? |
| If yes, what are the reasons for this decline or disappearance? |
| Have you heard about the recent Ebola outbreak affecting Guinea? |
| Are there animal species that you avoid eating traditionally? |
| Are there wildlife species that you have been avoiding eating during the Ebola outbreak? |
| Do you know these species (see photo in the appendix)? 1= Bat, 2 = Chimpanzees; 3 = Duiker; 4 = Monkey; 5 = Crested porcupine, 6 = Greater cane rat; 7= Warthog |
| Are these species (see photo in the appendix) present in your area? 1= Bat, 2 = Chimpanzees; 3 = Duiker; 4 = Monkey; 5 = Crested porcupine, 6 = Greater cane rat; 7= Warthog |
| Which of the animals mentioned above are currently being consumed? Do you currently consume these species? |
| Were these also consumed before the Ebola epidemic? Which ones were consumed before the Ebola outbreak? Which species did you consume before the Ebola epidemic? |
| Have you changed your livestock meat consumption since the Ebola outbreak? Source (market, own animals or other)? If More or Less, ask why? |
| Have you changed your fish consumption during the Ebola outbreak? Source (market, own fishing or other)? If More or Less, ask why? |
| Do you know if there are any registered cases of Ebola in this area? |
| What are your sources of information regarding Ebola disease? |
| What measures do you know of to fight/avoid Ebola virus? |
| What is your perception about these animal groups (1= Bat, 2 = Chimpanzees; 3 = Duiker; 4 = Monkey; 5 = Crested porcupine, 6 = Greater cane rat; 7= Warthog) as potential carriers of the Ebola virus? |

**II- Local restaurant owners or manager/ Bushmeat sellers**

| **Questions** |  |
| --- | --- |
| How many years have you been a bushmeat seller/ restaurant manager/owner? |  |
| What are the main species of fauna in this area? |  |
| Which animals do you use as bushmeat for cooking? |  |
| Are there animal species that you avoid eating traditionally? |  |
| Are there wildlife species that you have been avoiding eating during the Ebola outbreak? |  |
| Do you know these species (see photo in the appendix)? 1= Bat, 2 = Chimpanzees; 3 = Duiker; 4 = Monkey; 5 = Crested porcupine, 6 = Greater cane rat; 7= Warthog |  |
| Are these species (see photos in the appendix) present in your area? 1= Bat, 2 = Chimpanzees; 3 = Duiker; 4 = Monkey; 5 = Crested porcupine, 6 = Greater cane rat; 7= Warthog |  |
|  |  |
| Which of the animals mentioned above are currently being consumed? Do you currently consume these species? |  |
| Were these also consumed before the Ebola epidemic? Which ones were consumed before the Ebola outbreak? Which species did you consume before the Ebola epidemic? |  |
| Have you changed your livestock meat consumption since the Ebola outbreak? Source (market, own animals or other)? If More or Less, ask why? |  |
| Have you changed your fish consumption during the Ebola outbreak? Source (market, own fishing or other)? If More or Less, ask why? |  |
| Which are the species most appreciated by your customers? |  |
| Do you know any species that are rare today or have disappeared in this area? Why? |  |
| What difference do you make between the price of bushmeat and that of livestock meat? Why this difference? |  |
| Has the price of bushmeat changed during the Ebola outbreak? |  |
| Has the price of fish changed during the Ebola outbreak? |  |
| Do you know of any registered cases of Ebola in this area? |  |
| What are your sources of information regarding the Ebola disease? |  |
| What measures do you know of to fight/avoid Ebola virus? |  |
| What is your perception about these animal groups (1= Bat, 2 = Chimpanzees; 3 = Duiker; 4 = Monkey; 5 = Crested porcupine, 6 = Greater cane rat; 7= Warthog) as potential carriers of the Ebola virus? |  |

1. **Farmer/ Housewives**

| **Questions** |
| --- |
| What are the types of vegetation found in this locality? |
| What are the main species of fauna in this area? |
| In your opinion, what animal species in this area have become rare or extinct? |
| Do wildlife forage on your crops? How do you feel about that? |
| Are there animal species that you avoid eating traditionally? |
| Are there wildlife species that you have avoided eating during the Ebola outbreak? |
| Do you know these species (see photo in the appendix)? 1= Bat, 2 = Chimpanzees; 3 = Duiker; 4 = Monkey; 5 = Crested porcupine, 6 = Greater cane rat; 7= Warthog |
| Are these species (see photos in the appendix) present in your area? 1= Bat,  2 = Chimpanzees; 3 = Duiker; 4 = Monkey;  5 = Crested porcupine, 6 = Greater cane rat; 7= Warthog |
| Which of the animals mentioned above are currently being consumed? Do you currently consume these species? |
| Were these also consumed before the Ebola epidemic? Which ones were consumed before the Ebola outbreak? Which species did you consume before the Ebola epidemic? |
| Have you changed your livestock meat consumption since the Ebola outbreak? Source (market, own animals or other)? If More or Less, ask why? |
|  |
| Have you changed your fish consumption since the Ebola outbreak? Source (market, own animals or other)? If More or Less, ask why? |
| Do you know of any registered cases of Ebola in this area? |
| What are your sources of information regarding Ebola? |
| What measures do you know of to fight/avoid Ebola virus? |
| What difference do you make between the price of bushmeat and that of livestock meat? Why this difference? |
| What is your perception about these animal groups (1= Bat, 2 = Chimpanzees; 3 = Duiker; 4 = Monkey; 5 = Crested porcupine, 6 = Greater cane rat; 7= Warthog) as potential carriers of the Ebola virus? |

**IV- Authorities/ Elder men**

| **Questions** |
| --- |
| What are the types of vegetation found in this locality? |
| What are the main species of fauna in this area? |
| In your opinion, what animal species in this area have become rare or extinct? |
| How do you appreciate the current status of the wildlife in this area? |
| What is the level of application of the texts, laws and legislation on wildlife conservation? |
| Does the forest administration carry out wildlife inventory activities in this area? |
| Are the local people involved in the management of natural resources? |
| Are there animal species that you avoid eating traditionally? |
| Are there wildlife species that you have been avoiding eating during the Ebola outbreak? |
| Do you know these species (see photo in the appendix)? 1= Bat, 2 = Chimpanzees; 3 = Duiker; 4 = Monkey; 5 = Crested porcupine, 6 = Greater cane rat; 7= Warthog |
| Are these species (see photos in the appendix) present in your area? 1= Bat, 2 = Chimpanzees; 3 = Duiker; 4 = Monkey; 5 = Crested porcupine, 6 = Greater cane rat; 7= Warthog |
|  |
| Which of the animals mentioned above are currently being consumed? Do you currently consume these species? |
| Were these also consumed before the Ebola epidemic? Which ones were consumed before the Ebola outbreak? Which species did you consume before the Ebola epidemic? |
| Have you changed your livestock meat consumption since the Ebola outbreak? Source (market, own animals or other)? If More or Less, ask why? |
| Have you changed your fish consumption since the Ebola outbreak? Source (market, own animals or other)? If More or Less, ask why? |
| Do you know of any registered cases of Ebola in this area? |
| What are your sources of information regarding Ebola: |
| What measures do you know of to fight/avoid Ebola virus? |
| What difference do you make between the price of bushmeat and that of livestock meat? Why this difference? |
| What is your perception about these animal groups (1= Bat, 2 = Chimpanzees; 3 = Duiker; 4 = Monkey; 5 = Crested porcupine, 6 = Greater cane rat; 7= Warthog) as potential carriers of the Ebola virus? |
